# Magnetization Transfer Ratio Relates to Cognitive Impairment in Normal Elderly

**DOI:** 10.3389/fnagi.2014.00263

**Published:** 2014-09-25

**Authors:** Stephan Seiler, Lukas Pirpamer, Edith Hofer, Marco Duering, Eric Jouvent, Franz Fazekas, Jean-Francois Mangin, Hugues Chabriat, Martin Dichgans, Stefan Ropele, Reinhold Schmidt

**Affiliations:** ^1^Department of Neurology, Medical University of Graz, Graz, Austria; ^2^Institute of Medical Informatics, Statistics and Documentation, Medical University of Graz, Graz, Austria; ^3^Institute for Stroke and Dementia Research, Klinikum der Universität München, Ludwig-Maximilians-University, Munich, Germany; ^4^Department of Neurology, CHU Lariboisière, Paris, France; ^5^Neurospin, CEA Saclay, Saclay, France; ^6^German Center for Neurodegenerative Diseases (DZNE), Munich, Germany; ^7^Munich Cluster for Systems Neurology (SyNergy), Munich, Germany

**Keywords:** cerebrovascular disease, dementia, cognitive aging, magnetic resonance imaging, magnetization transfer imaging, microstructural tissue damage

## Abstract

Magnetization transfer imaging (MTI) can detect microstructural brain tissue changes and may be helpful in determining age-related cerebral damage. We investigated the association between the magnetization transfer ratio (MTR) in gray and white matter (WM) and cognitive functioning in 355 participants of the Austrian stroke prevention family study (ASPS-Fam) aged 38–86 years. MTR maps were generated for the neocortex, deep gray matter structures, WM hyperintensities, and normal appearing WM (NAWM). Adjusted mixed models determined whole brain and lobar cortical MTR to be directly and significantly related to performance on tests of memory, executive function, and motor skills. There existed an almost linear dose-effect relationship. MTR of deep gray matter structures and NAWM correlated to executive functioning. All associations were independent of demographics, vascular risk factors, focal brain lesions, and cortex volume. Further research is needed to understand the basis of this association at the tissue level, and to determine the role of MTR in predicting cognitive decline and dementia.

## Introduction

Diffusion tensor imaging (DTI) and magnetization transfer imaging (MTI) are MRI-based methods enabling detection of microstructural changes in various brain tissue compartments. They allow to quantify cerebral brain damage beyond what can be expected from standard MRI techniques (O’Sullivan et al., 2004; Vernooij et al., 2009; Schmidt et al., 2010; Xu et al., 2010). While DTI measures the diffusion properties of tissue water (Mori and Zhang, 2006). MTI is based on the exchange of magnetization between tissue water and protons bound to macromolecules (Graham and Henkelman, 1997). Histopathological correlations demonstrated the association of lower magnetization transfer ratios (MTR) with decreasing myelin content and axonal count in both the white matter (WM) and cortex (Schmierer et al., 2007; Chen et al., 2013). In a previous study of our own group, we found a significant MTR decrease in normal appearing white matter (NAWM) and cortex with advancing age (Fazekas et al., 2005). The age-related effect was stronger in the cortex than in NAWM. The effect was strongest in frontal and parieto-occipital cortical regions (Fazekas et al., 2005). Draganski et al. (2011) replicated the age-dependent MTR decrease within the frontal cortex in a study on 26 healthy adults, aged 18–85 years. Additionally, these authors described age-associated MTR changes within the basal ganglia and cerebellar cortex. While there exists convincing evidence that DTI measures in NAWM correlate with cognitive functioning in normal aging (Charlton et al., 2006; Vernooij et al., 2009), and various disease states (Huang and Auchus, 2007; Chen et al., 2009; Xu et al., 2010; Zhou et al., 2011), little is known about the clinical importance of MTR metrics. Previous studies reported conflicting results. One investigation in 64 healthy subjects aged 50–90 years (Schiavone et al., 2009) reported that lower MTR in NAWM related to impairment of processing speed, executive function, and episodic memory. Conversely, two other studies failed to demonstrate any significant association between both cortical- or WM MTR and cognitive function in healthy subjects (Fazekas et al., 2005; Deary et al., 2006).

It is important to note that cortical MTR reductions have also been described in Alzheimer’s disease (AD) patients. MTR changes followed an AD-specific pattern even after adjustment for cortical atrophy (Hanyu et al., 2000; Ridha et al., 2007; Giulietti et al., 2012). Lower MTR in the cortex of AD patients related to scores on the mini mental state examination (MMSE). Significant direct associations were described with temporal cortex MTR (van Es et al., 2007) and with deep gray matter MTR (Ropele et al., 2012).

The current investigation examined a large cohort of 355 community-dwelling subjects over a wide age range. We hypothesized that MTR scales with the severity of tissue changes and relates to worse cognitive performance even when controlling for vascular risk factors and brain abnormalities on conventional MRI. We also hypothesized that the regional distribution of MTR changes determines the pattern of cognitive impairment.

## Materials and Methods

### Subjects

The study cohort is from the Austrian stroke prevention family study (ASPS-Fam), a prospective single-center, community-based study on the cerebral effects of vascular risk factors in the normal elderly population of the city of Graz, Austria. The ASPS-Fam represents an extension of the Austrian stroke prevention study (ASPS), which was established in 1991 (Schmidt et al., 1994, 1999). Between 2006 and 2013, study participants of the ASPS and their first grade relatives were invited to enter ASPS-Fam. Inclusion criteria were no history of previous stroke or dementia and a normal neurologic examination. A total of 381 individuals from 169 families were included into the study. The number of members per family ranged from two to six.

The entire cohort underwent an extended diagnostic work-up including clinical history, blood tests, cognitive testing, and a thorough vascular risk factor assessment. All individuals underwent MRI, except for 26 who had contraindications. Thus, MTI was available in a total of 355 subjects. The study protocol was approved by the ethics committee of the Medical University of Graz, Austria, and written informed consent was obtained from all subjects.

### Vascular risk factors

Assessment of vascular risk factors included arterial hypertension, diabetes mellitus, cardiac disease, hypercholesterolemia, hypertriglyceridemia, hyperuricemia, peripheral vascular disease, and venous thrombotic disease and was determined based on history and measurements at the examination as previously described (Schmidt et al., 1999).

### Neuropsychological testing

A test battery assessing memory, executive function, and motor skills was administered as described previously (Schmidt et al., 1999). Briefly, the tests employed have been widely used in the German-speaking area and were always applied in the same order and under same laboratory conditions. Intermediate memory recall and learning ability was assessed by the “Bäumler’s Lern- und Gedächtnistest” (LGT-3) (Bäumler, 1979), a highly demanding paper–pencil procedure consisting of six subtests. Three subtests (word and digit association tasks, and story recall) screen for verbal memory, and two subtests (trail and design recall) screen for visuospatial memory. The sum of weighted scores from these subtests and of an image recognition paradigm result in the total learning and memory performance score. The stimulus sets of the word association task (German-Turkish word pairs), the story (facts about construction of a library), and design recall (core symbol and frame), and the recognition paradigm (objects) consist of 20 items each. A trail in an abstracted city map serves as the trail recall test. These sets of stimuli were presented to the person being tested for 1 min. Two minutes were given for learning the 13 items of the digit association task (three-digit telephone numbers and names of extension holders). During a learning phase, the six sets of stimuli are subsequently presented to the person being tested. The recall phase starts immediately thereafter and follows the same order. The delay between presentation and recall for a given subtest ranges between 7 and 11 min. Executive functions were tested by the Wisconsin card sorting test (Heaton, 1981), part B of the trail making test (United States War Department, 1944), and digit span backwards, which is part of the Wechsler adult intelligence scale, revised (Tewes, 1991). Adhering to Milner’s criteria (Milner, 1963), the measures computed for the Wisconsin card sorting test were categories completed, perseverative errors, and total errors.

Motor skills were evaluated by the Purdue pegboard test (Tiffin and Asher, 1948).

To reduce floor and ceiling artifacts and other sources of measurement error, we used summary measures of cognitive function in the analyses rather than the results of individual tests. We formed composite measures of the specific domains of cognitive function. Each summary measure was calculated by converting individual test scores to *z*-scores within the group and by computing the average of the scores in each cognitive domain.

### Magnetic resonance imaging

Magnetic resonance imaging was performed on a 3T whole body scanner (TimTrio; Siemens Healthcare, Erlangen, Germany) and included conventional imaging and MTI. The MT sequence was based on a spoiled 3D gradient-echo sequence (TR = 40 ms, TE = 7.38 ms, flip angle = 15°, number of slices = 40, slice thickness = 3 mm, in-plane resolution = 0.86 mm × 0.86 mm) that was performed with and without a Gaussian shaped MT saturation pulse.

The conventional protocol included an axial FLAIR sequence (TR = 10000 ms, TE = 69 ms, inversion time = 2500 ms, number of slices = 40, slice thickness = 3 mm, in-plane resolution = 0.86 mm × 0.86 mm) and a high resolution T1 weighted 3D sequences with magnetization preparation (MPRAGE) and whole brain coverage (TR = 1900 ms, TE = 2.19 ms, inversion time = 900 ms, flip angle = 9°, isotropic resolution of 1 mm).

White matter hyperintensities (WMH), silent non-lacunar, and lacunar infarcts were recorded on FLAIR images as previously described (Schmidt et al., 1999). Non-lacunar infarcts were lesions with typical signal characteristics of infarcts following a typical vascular territory or being located in a border zone between two vascular territories. Lacunes were focal lesions involving the basal ganglia, the internal capsule, the thalamus, the brainstem, or the WM not exceeding a maximum diameter of 20 mm. All lesions were outlined using a custom written IDL program (Exelis Visual Information Solutions, USA). Lesion areas were segmented by combined region growing and local thresholding following manual selection (Plummer, 1992). The total lesion volume (cubic millimeter) was calculated using the program FSLMATHS (FSL, Oxford^1^) by multiplying the lesion area with the slice thickness and normalized by head size. Cortex volume, normalized for the subject head size, was calculated from the T1 weighted MPRAGE images using the fully automated structural image evaluation of atrophy (FSL, Oxford^1^).

### Image processing and MTR analysis

Magnetization transfer ratio metrics were assessed separately for the cortex, deep gray matter structures, WMH, and NAWM. MTR maps were calculated using the formula MTR = (Mss − M0)/M0, where Mss and M0 are the signal intensities obtained with and without MT saturation, respectively. The MTR maps were registered to the corresponding FLAIR and T1 scans by using an automated affine registration tool (FLIRT as part of FSL^2^ ). WMH masks were generated from the outlined lesions using our custom written IDL tool (Plummer, 1992). A mean MTR was calculated in MTR space for each WMH by masking the registered MTR maps with the WMH masks. The MTRs of all WMHs were averaged to obtain a mean lesion MTR for each subject’s whole brain-, periventricular-, and deep WMH.

White matter and cortex masks were generated using the program FAST (FSL, Oxford^1^). Both NAWM and cortex masks were generated separately and comprised all WM and cortical tissue outside WMHs, silent non-lacunar infarcts and lacunes, i.e., it comprised all neocortical gray matter and WM with normal signal intensity on FLAIR images. Corresponding MTR maps were produced by overlying both the cortex and NAWM masks separately on the MTR maps and a mean MTR for total NAWM and cortex was calculated for each subject. Calculation of lobar MTRs comprising frontal, parietal, occipital, and temporal lobes was done by applying a home-written atlas tool. The atlas tool was generated in MNI 152 space by manually delineating neuroanatomical borders of the four lobes. The atlas was then registered to the respective patient’s native space and overlaid on the total NAWM/cortex MTR maps to obtain lobar MTR values.

Deep gray matter structures including the thalamus, putamen, pallidum, hippocampus, caudate nucleus, amygdala, and accumbens nucleus were generated using the program FIRST (FSL, Oxford^1^). Corresponding MTR maps were produced by overlying the deep gray matter structure masks on the MTR maps and subsequently a mean MTR of each structure was calculated for each subject. To reduce partial volume effects, which might have occurred due to image registration and subsequent interpolation, we eroded all masks by 1 pixel before overlying on the MTR maps.

### Statistical analysis

Assumptions of normal distribution were tested with the Kolmogorov–Smirnov test. Normally distributed variables are reported as mean ± SD and non-normally distributed variables as median and interquartile range. For bivariate correlations, Pearson’s correlation analysis has been performed. Due to the bimodal distribution of age (Figure S1 in Supplementary Material), we used age as an ordinal variable categorized into three categories (38–60 years; 61–70 years; 71–86 years) in all statistical models.

White matter hyperintensities volume had a skewed distribution containing zero values and therefore the value 2 was added to the volumes before natural log transformation.

Multivariate linear and logistic regression analyses were used to correlate visible brain changes (WMH volume, presence of silent non-lacunar infarcts, presence of lacunes) with global and regional MTR metrics in the cortex and NAWM. These models were adjusted for age, sex, vascular risk factors, and normalized cortex volume. We use cortex volume as a covariate because it is widely accepted that it strongly correlates with cognitive changes across the aging and dementia spectrum. To assess the associations between global and regional mean MTRs in the cortex, NAWM and WMH, and domain-specific neuropsychological test performance, mixed models were calculated. In addition, age- and sex stratified sub analyses were done. The cognitive variable was the dependent and MTR was the predictor variable. These models were adjusted for potential confounders to evaluate the independent effect of MTR on cognitive functions. Selection of confounders was *a priori* defined according to previous literature (van der Flier et al., 2005; Verdelho et al., 2010). We considered age, sex, education, vascular risk factors, silent non-lacunar infarcts, lacunes, WMH volume, and cortex volume as possible confounders. Multicollinearity was assessed between the independent variables of the models using the variance inflation factor (VIF). A VIF value >10 is an indicator of multicollinearity. There was no indication for multicollinearity in the models. The mean VIF for predictor variables in our study was 1.34 (range 1.03–3.48).

To account for the sample relationships in current family based study, a random effect was added to each model using a kinship matrix describing the family structure (Newton-Cheh et al., 2009; Suchindran et al., 2009).

We corrected the mixed model analyses and sub-analyses for multiple comparisons within each domain using the Benjamini–Hochberg false discovery rate (FDR, *p* < 0.05) (Benjamini and Hochberg, 1995).

To determine possible dose-effect relationships of significant associations between MTR and cognitive functioning, subjects were categorized into quartiles according to MTR value distribution. Linear regression analyses with MTR quartiles being the explanatory variables and cognitive test results being the dependent variable adjusted for age, sex, education, risk factors, lacunes, non-lacunar infarcts, WMH, and normalized cortex volume were done. The highest quartile of MTR values served as the reference.

For each regression coefficient, the 95% confidence interval and the *p*-value were determined. A two-sided *p*-value <0.05 was considered to be statistically significant.

The multicollinearity check and *t*-tests were performed with SPSS (IBM Statistics for Windows, Version 19, Armonk, NY, USA). The mixed models and the kinship matrices were calculated using the coxme^3^ and kinship2^4^ packages in R (R: a language and environment for statistical computing, Vienna, Austria^5^ ). We assessed if age effects on cognitive function were mediated by MTR variables. Estimation of mediator effect sizes was calculated using bootstrapped models (Hayes and Preacher, 2013). According to Preacher and Hayes (2008), significant mediation can be determined via the 95% bootstrapped confidence interval. If “zero” lies within the interval range, it is possible with 95% confidence that the true indirect effect would be zero (no mediation). If “zero” does not occur between the interval boundaries then we can conclude that the indirect effect for this mediator is significant (Preacher and Hayes, 2008). Mediation was assessed for cortical and NAWM MTR individually.

## Results

Demographics, frequency of vascular risk factors, neuropsychological test results, and MRI findings of the study participants are displayed in Table 1. Lacunes and non-lacunar infarcts were inversely related to cortical MTR (β = −0.55; 95% CI [−1.003;−0.112], *p* = 0.014 and β = −0.53; 95% CI [−0.890;−0.170], *p* = 0.003), and NAWM MTR (β = −0.603; 95% CI [−0.958;−0.248], *p* = 0.0009 and β = −0.44; 95% CI [−0.900;0.010], *p* = 0.06) after adjustment for age, sex, and vascular risk factors.

**Table 1:** **Demographics, risk factors, neuropsychological test performance, and MRI findings of study participants**.

**A. BASIC DEMOGRAPHICS**	
Age, years (median, IQR)	68.00 (56.00–72.00)
Age category 1: 38–60 years, *N* (%)	100 (28.2)
Age category 2: 61–70 years, *N* (%)	131 (36.9)
Age category 3: 71–86 years, *N* (%)	124 (34.9)
Women *N* (%)	214 (60.3)
Education *N* (%)	
Primary school *N* (%)	70 (19.7)
Apprenticeship *N* (%)	157 (44.2)
High school diploma *N* (%)	72 (20.3)
University degree *N* (%)	56 (15.8)
**B. RISK FACTORS**	
Arterial hypertension *N* (%)	229 (64.5)
Diabetes *N* (%)	38 (10.7)
Heart disease *N* (%)	186 (52.4)
Hypercholesterolemia *N* (%)	272 (76.6)
Hypertriglyceridemia *N* (%)	60 (16.9)
Hyperuricemia *N* (%)	104 (29.3)
Peripheral vascular disease *N* (%)	5 (1.4)
Venous embolic disease *N* (%)	38 (10.7)
**C. NEUROPSYCHOLOGICAL TESTING (*z*-VALUES)**	
Memory, *z*-values (range)	−1.14–3.51
Executive function, *z*-values (range)	−4.15–1.35
Motor skills, *z*-values (range)	−2.49–3.12
**D. MRI VARIABLES**	
Lacunes *N* (%)	33 (9.4)
Silent non-lacunar infarcts *N* (%)	19 (5.5)
Cortex volume, cubic centimeter (mean ± SD)	599.69 (40.11)
WMH volume, cubic centimeter (median, IQR)	5.63 (2.97–10.76)

*MRI, magnetic resonance imaging, WMH, white matter hyperintensities, SD, standard deviation, IQR, interquartile range*.

There existed no significant association between WMH volume and cortical or NAWM MTR (β = 0.015; 95% CI [−0.037;0.067], *p* = 0.570 and β = −0.002, 95% CI [0.054;0.050], *p* = 0.940, respectively).

Magnetization transfer ratio of cortex and NAWM correlated significantly and independently of subject characteristics and MRI findings with each other (β = 0.68; 95% CI [0.594;0.757], *p* < 0.001).

There existed a strong correlation between cortical MTR and cognitive functions (Figure S2 in Supplementary Material).

Table 2 displays the independent relationship between cortical MTR and cognitive functioning. As can be seen from this table, whole brain, and all lobar cortical MTRs were significantly and directly associated with performance in memory and executive function. For motor skills only the temporo-occipital MTRs were significantly related and there was a non-significant trend for fronto-parietal lobe MTR. The MTR of deep gray matter structures correlated with executive function, but not with scores of memory and motor skills. Stratified analyses by tertiles of age showed no age-group specific association between cortical and lobar MTR for any cognitive domain with the exception of motor skills for which a significant relationship existed only in the highest age-group (data not shown).

**Table 2:** **Multivariate linear regression analysis^a^: cortical/deep gray matter MTR and cognitive functioning**.

Mean MTR	Memory			Executive function			Motor skills		
	β	95% CI	*p*	β	95% CI	*p*	β	95% CI	*p*
**CORTEX**									
Whole brain	0.129	[0.038;0.220]	0.0366	0.118	[0.051;0.192]	0.0044	0.111	[0.033;0.189]	0.0418
Frontal lobe	0.119	[0.030;0.209]	0.0366	0.116	[0.049;0.188]	0.0044	0.097	[0.020;0.173]	0.0616
Parietal lobe	0.129	[0.042;0.217]	0.0366	0.123	[0.058;0.194]	0.0033	0.095	[0.020;0.171]	0.0616
Occipital lobe	0.121	[0.035;0.208]	0.0366	0.129	[0.066;0.199]	0.0022	0.104	[0.030;0.178]	0.0418
Temporal lobe	0.108	[0.022;0.194]	0.0409	0.086	[0.022;0.155]	0.0229	0.111	[0.038;0.184]	0.0418
**DEEP GRAY MATTER**									
Thalamus	0.023	[−0.027;0.073]	0.4515	0.044	[0.006;0.082]	0.0421	0.038	[−0.008;0.078]	0.2514
Putamen	0.033	[−0.020;0.085]	0.3457	0.057	[0.017;0.097]	0.0183	0.036	[−0.013;0.077]	0.3025
Pallidum	0.023	[−0.022;0.068]	0.4262	0.034	[−0.001;0.068]	0.0864	0.045	[0.007;0.084]	0.0770
Caudate nucleus	0.024	[−0.025;0.074]	0.4400	0.062	[0.024;0.100]	0.0057	0.027	[−0.016;0.070]	0.4400
Amygdala	0.018	[−0.023;0.060]	0.4515	0.029	[−0.003;0.061]	0.1173	0.019	[−0.017;0.055]	0.5316
Accumbens nucleus	−0.011	[−0.046;0.024]	0.5830	0.020	[−0.007;0.047]	0.1941	0.011	[−0.019;0.041]	0.5988

*^a^Adjusted for age, sex, years of education, vascular risk factors, cortex volume, thromboembolic infarcts, lacunes, and WMH volume*.

*MTR, magnetization transfer ratio; *p*-values adjusted for multiple testing*.

Table 3 displays the associations between MTR in NAWM and cognitive status. Significant direct relationships were found with memory and executive function. This was seen for NAWM MTR in the whole brain and for NAWM MTR in all lobes with the exception of the temporal lobe.

**Table 3:** **Multivariate linear regression analysis^a^: normal appearing white matter (NAWM) and cognitive functioning**.

Mean NAWM MTR	Memory			Executive function			Motor skills		
	β	95% CI	*p*	β	95% CI	*p*	β	95% CI	*p*
Whole brain	0.112	[0.022;0.202]	0.0412	0.085	[0.015;0.155]	0.0340	0.030	[−0.048;0.108]	0.5988
Frontal lobe	0.100	[0.016;0.184]	0.0462	0.073	[0.007;0.139]	0.0524	0.023	[−0.050;0.096]	0.6252
Parietal lobe	0.121	[0.033;0.209]	0.0366	0.091	[0.022;0.160]	0.0229	0.032	[−0.044;0.108]	0.5988
Occipital lobe	0.114	[0.027;0.202]	0.0366	0.094	[0.026;0.161]	0.0201	0.047	[−0.028;0.122]	0.4400
Temporal lobe	0.062	[−0.015;0.139]	0.2200	0.036	[−0.024;0.096]	0.2778	0.047	[−0.019;0.113]	0.3911

*^a^Adjusted for age, sex, years of education, vascular risk factors, cortex volume, thromboembolic infarcts, lacunes, and WMH volume*.

*NAWM, normal appearing white matter, MTR, magnetization transfer ratio; *p*-values adjusted for multiple testing*.

Lower mean MTR in WMH related to poorer performance in executive function tests (β = 0.038; 95% CI [0.008;0.069], *p* = 0.033). The associations with test results in the other cognitive domains were not significant.

Mixed model analysis stratified by sex demonstrated that the relationship between MTR in the cortex and memory as well as executive function scores was significant only in men but not in women (Table S1 in Supplementary Material).

**Figure 1:**
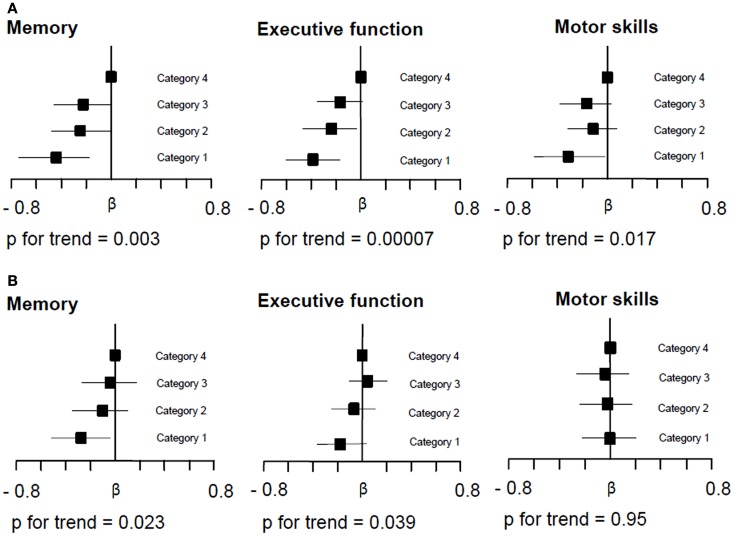
**Relationship between whole brain cortical (A) and normal appearing white matter (NAWM) MTR (B) and domain-specific cognitive performance**. Multiple regression analysis adjusted for age, sex, educational level, vascular risk factors, cortex volume, silent non-lacunar infarcts, lacunes, and white matter hyperintensity volume compares the effect between MTR quartiles on performance on tests of memory, executive function, and motor skills with the highest quartile of the MTR distribution serving as the reference. The range of neocortical MTR values in quartiles 1, 2, 3, and 4 was 22.98–28.16, 28.17–28.94, 28.95–29.46, and 29.47–33.06, respectively. The range of NAWM MTR values in quartiles 1, 2, 3, and 4 was 34.25–39.23, 39.24–39.81, 39.82–40.36, and 40.37–44.70, respectively. Squares on the *x*-axis indicate the β-coefficients and bars give the 95% confidence intervals. **(A)** Demonstrates that decreasing MTR in whole brain cortical MTR related to poorer performance in memory, executive function, and motor skills tests. With decreasing MTR quartile distribution there was an almost linear decline in memory and executive function performance. The association for motor skills was non-linear. **(B)** Demonstrates that also decreasing whole brain NAWM MTR was significantly related to memory performance and executive function. The relative dose-dependent effect of MTR in NAWM was less pronounced than that seen for cortical MTR.

Figure 1 demonstrates the associations between quartiles of mean MTR distribution in the cortex and in NAWM of the whole brain and domain-specific cognitive test results. There was a significant almost linear relationship between decreasing cortical MTR values and impairment in memory and executive function. The association with motor skills was of borderline significance and it was non-linear. For NAWM MTR a significant dose-effect relationship existed for memory performance and executive function. When assessing a possible mediating effect of cortex and NAWM MTR on the relationship between age and cognition, we found a significant mediation of cortical and NAWM MTR on the relationship between age and executive function, but not on memory or on motor skills (Table 4).

**Table 4:** **Analysis of mediating effects of MTR variables on the relationship between age and cognitive test results**.

	Total effect	Direct effect	Indirect effect	Bootstrapped CI
**MTR CORTEX WHOLE BRAIN**				
Memory	−0.0395	−0.0344	−0.0051	[−0.0114;0.0006]
Executive function	−0.0211	−0.0157	−0.0055*	[−0.0103;−0.0016]
Motor skills	−0.0408	−0.0371	−0.0036	[−0.0084;0.0005]
**MTR NAWM WHOLE BRAIN**				
Memory	−0.0397	−0.0363	−0.0034	[−0.0081;0.0008]
Executive function	−0.0211	−0.0183	−0.0027*	[−0.0059;−0.0006]
Motor skills	−0.0413	−0.0414	0.0001	[−0.0026;0.0031]

*Predictor variable = age; outcome variable = memory, executive function, motor skills; mediator variable = MTR cortex whole brain, MTR NAWM whole brain*.

*MTR, magnetization transfer ratio; NAWM, normal appearing white matter. Adjusted for age, sex, years of education, vascular risk factors, cortex volume, thromboembolic infarcts, lacunes, and WMH volume*.

Significant indirect effects are marked (*)

## Discussion

In this large family-based study in community-dwelling subjects without a history of clinical strokes or signs of dementia, we found that lower whole brain and lobar MTR in the cortex is related to memory impairment, executive dysfunction, and impaired motor skills. The associations were more pronounced in men than in women. There was an almost linear dose-effect relationship between the quartile distribution of MTR in the cortex and performances in all cognitive domains. Lowering of MTR in deep gray matter related to executive dysfunction, which is in line with the hypothesis that the regional pattern of MTI-detected microstructural tissue alterations determines the pattern of cognitive impairment. Executive dysfunction occurs with lesions involving frontal-subcortical circuits (Bonelli and Cummings, 2007; Duering et al., 2011; Krause et al., 2012). These circuits originate in the prefrontal cortex, project to the striatum, connect to the globus pallidus and substantia nigra, and from there to the thalamus (Alexander et al., 1986; Purves et al., 2001).

Notably, the association between MTR in NAWM and cognitive performance was much weaker than that observed between MTR in the cortex and neuropsychological test results. The consistent direct relationship between cortex MTR and cognition in this study in middle aged and elderly persons, which was independent of the presence and extent of structural brain changes and normalized cortex volume suggests that widespread microstructural tissue changes in the cortex are an important early substrate of age-related cognitive decline. The cause for diffuse cortical MTR decrease during brain aging remains to be determined.

We realize that diffuse cortical MTR reductions might simply reflect partial volume effects from CSF as a consequence of age-related loss of brain parenchyma. CSF has MTR values approaching zero. Increased sulcal widening could thus result in a decrease of MTR in voxels with mixed contributions from cortex and CSF. Several points argue against this explanation. While the slice thickness of the MT sequence used in the current study was only moderately thin, it had an in-plane resolution higher than the hires T1 scan that was used for segmentation of the cortex. All analyses were corrected for cortical volume (Rashid et al., 2004; Takao et al., 2011; Metzler-Baddeley et al., 2012). Moreover, all MTR masks in our study have been eroded by one voxel to avoid inclusion of “edge” values, which should make CSF contamination of cortex MTR unlikely. It is also of note, that an MTR decrease was associated with cognitive impairment not only in neocortical but also in deep gray matter structures. Other than surface gray matter, which is surrounded by CSF, deep gray matter structures are surrounded by WM, which in case of partial volume effects would have rather increased MTR values. We can only speculate on the histological substrates that may lead to MTR lowering in the aging brain. So far, histopathological correlations with MTR were only done in post-mortem brain tissue of patients with multiple sclerosis (Petzold et al., 2011; Chen et al., 2013). MTR was considered to primarily reflect the amount of myelin (Barkhof et al., 2003; Schmierer et al., 2007) in WM structures, but also axonal count (Schmierer et al., 2007). A recent study also reported MTR lowering with focal demyelination in the MS cortex (Chen et al., 2013).

In the aging brain demyelination often occurs as a consequence of cerebral small vessel disease (Grinberg and Thal, 2010; Pantoni, 2010; Schmidt et al., 2011). If demyelination in the wake of cerebral small vessel disease was indeed the leading cause for the MTR changes seen in our study, we would have expected to find an inverse relationship between MTR values in different tissue compartments and WMH volume. This was neither the case for cortical MTR nor for NAWM MTR. Thus, other yet unknown microstructural changes might have been responsible. A recent study scanned unfixed post-mortem brain slices of 12 multiple sclerosis patients by MTI at 1.5 T, assessed blocks containing non-lesional brain tissue microscopically, and microdissected adjacent tissue to quantify specific protein levels (Petzold et al., 2011). The authors reported that post-translational modifications of axonal proteins such as phosphorylation of neurofilaments occurred in non-lesional brain tissue and suggested that the resulting lowering of MTR was caused by a hyperphosphorylation-related change in proton mobility. Several mechanisms driving neurodegeneration such as glutamate excitotoxicity or mitochondrial failure relate to Ca^2+^ influx, activation of kinases and subsequent protein phosphorylation (Brownlees et al., 2000; Akassoglou and Strickland, 2002). Altered cortical MTR in the aging brain may thus reflect hyper-phosphorylation of proteins and pathologic accumulation of soluble and non-soluble proteins, a process that is known to precede cell death in many primary degenerative diseases (Nakamura and Lipton, 2009). In this context, a recent MTI study in Alzheimer dementia and mild cognitive impairment is of interest (Wiest et al., 2012). The authors applied a model-based multiparameter approach that allowed to separately quantify the presence and amount of macromolecules in pre-specified regions of interest and to investigate the coupling characteristics of protons by modeling deposition and interactions between macromolecules (Wiest et al., 2012). MTI metrics indicating increased coupling to the environment which can be expected under conditions of pathologic protein accumulation differentiated patients with AD and MCI from controls with high accuracy. This suggests that MTI metrics including MTR can depict changes in the macromolecular tissue composition caused by neurodegenerative disease. This could be an explanation for our finding that a drop in MTR of gray matter had much stronger effects on cognitive functioning than MTR lowering in the WM compartment.

Cortical and NAWM MTR mediated the association between age and executive function in our analysis, but not between age and memory or motor skills. Such mediating effects between age and executive function have been previously described for DTI measures in NAWM of elderly persons (Kerchner et al., 2012; Salami et al., 2012).

Our study has several strengths. It is the largest cohort study using MTI to date. The study is community-based with prospectively planned radiological and clinical protocols. High scan resolution allowed segmentation of cortical and WM compartments. A limitation of our investigation is its cross sectional design. The variability of MTR occurring in asymptomatic individuals is relatively low, but despite that the associations between cortical MTR and cognitive performance were substantial with little regional variability.

At this point, the importance of MTI in a clinical setting is unknown. If a decrease in MTR indeed reflects alterations of the macromolecular tissue composition resulting from age-related neurodegenerative processes, MTI metrics may turn out to be very early markers of neurodegenerative processes during brain aging. There has been only a single small longitudinal MTI study in Alzheimer patients, which showed a progressive decrease of MTR paralleling cognitive decline over a one year follow-up period (Ropele et al., 2012). Longitudinal data in cognitively healthy subjects or in cases with prodromal dementia are not yet available. Such studies are warranted to determine the rate of MTR change over time during normal and pathologic brain aging and to assess the role of MTR as possible imaging biomarkers in dementia research.

## Author Contributions

Design and conceptualization of the study: Marco Duering, Eric Jouvent, Franz Fazekas, Jean-Francois Mangin, Hugues Chabriat, Martin Dichgans, Stefan Ropele, and Reinhold Schmidt. Analysis and interpretation of the data: Stephan Seiler, Lukas Pirpamer, Edith Hofer, Stefan Ropele, and Reinhold Schmidt. Drafting the manuscript: Stephan Seiler, Lukas Pirpamer, Edith Hofer, Marco Duering, Eric Jouvent, Franz Fazekas, Jean-Francois Mangin, Hugues Chabriat, Martin Dichgans, Stefan Ropele, and Reinhold Schmidt. Revising the work critically: Stephan Seiler, Lukas Pirpamer, Edith Hofer, Marco Duering, Eric Jouvent, Franz Fazekas, Jean-Francois Mangin, Hugues Chabriat, Martin Dichgans, Stefan Ropele, and Reinhold Schmidt. Agreement to be accountable for all aspects of the work: Stephan Seiler, Lukas Pirpamer, Edith Hofer, Marco Duering, Eric Jouvent, Franz Fazekas, Jean-Francois Mangin, Hugues Chabriat, Martin Dichgans, Stefan Ropele, and Reinhold Schmidt.

## Conflict of Interest Statement

Stephan Seiler, Lukas Pirpamer, Edith Hofer, Marco Duering, Eric Jouvent, Franz Fazekas, Jean-Francois Mangin, and Hugues Chabriat report no disclosures. Martin Dichgans reports grants from EU FP 7, grants from DFG/DLR, grants from BMBF, grants from Vascular Dementia Research Foundation, grants from Jackstaedt Foundation, grants from Corona Foundation, during the conduct of the study; personal fees from Bayer Vital GmbH, personal fees from Boehringer Ingelheim Pharma GmbH & Co. KG, personal fees from Biologische Heilmittel Heel GmbH, personal fees from Bristol-Myers Squibb GmbH & Co. KGaA, personal fees from Ever Neuro Pharma, personal fees from Lundbeck GmbH, personal fees from Sanofi-Aventis Deutschland GmbH, personal fees from Shire Deutschland GmbH, personal fees from DZNE e.V., personal fees from Georg Thieme Verlag GmbH, personal fees from UpToDate, personal fees from W. Kohlhammer, outside the submitted work; and Research Funding Industry: Bayer Vital GmbH, Eisai Medical Research Inc., Eisai Ltd., Essex Parhma GmbH, Ferrer Internacional, S.A., ICON Clinical Research GmbH. Stefan Ropele and Reinhold Schmidt have nothing to disclose.

## Supplementary Material

The Supplementary Material for this article can be found online at http://www.frontiersin.org/Journal/10.3389/fnagi.2014.00263/abstract

## Footnotes

^1^http://www.fmrib.ox.ac.uk

^2^http://www.fmrib.ox.ac.uk/fsl/flirt/index.html

^3^http://CRAN.R-project.org/package=coxme

^4^http://CRAN.R-project.org/package=kinship2

^5^http://www.R-project.org
