# Supplementary material for: Magnetization Transfer Ratio Relates to Cognitive Impairment in Normal Elderly
Source: Front Aging Neurosci. 2014 Sep 25;6:263. doi: 10.3389/fnagi.2014.00263 (PMC4174770; doi:10.3389/fnagi.2014.00263)
Supplement: Supplementary file 1 [file Presentation1.PDF]

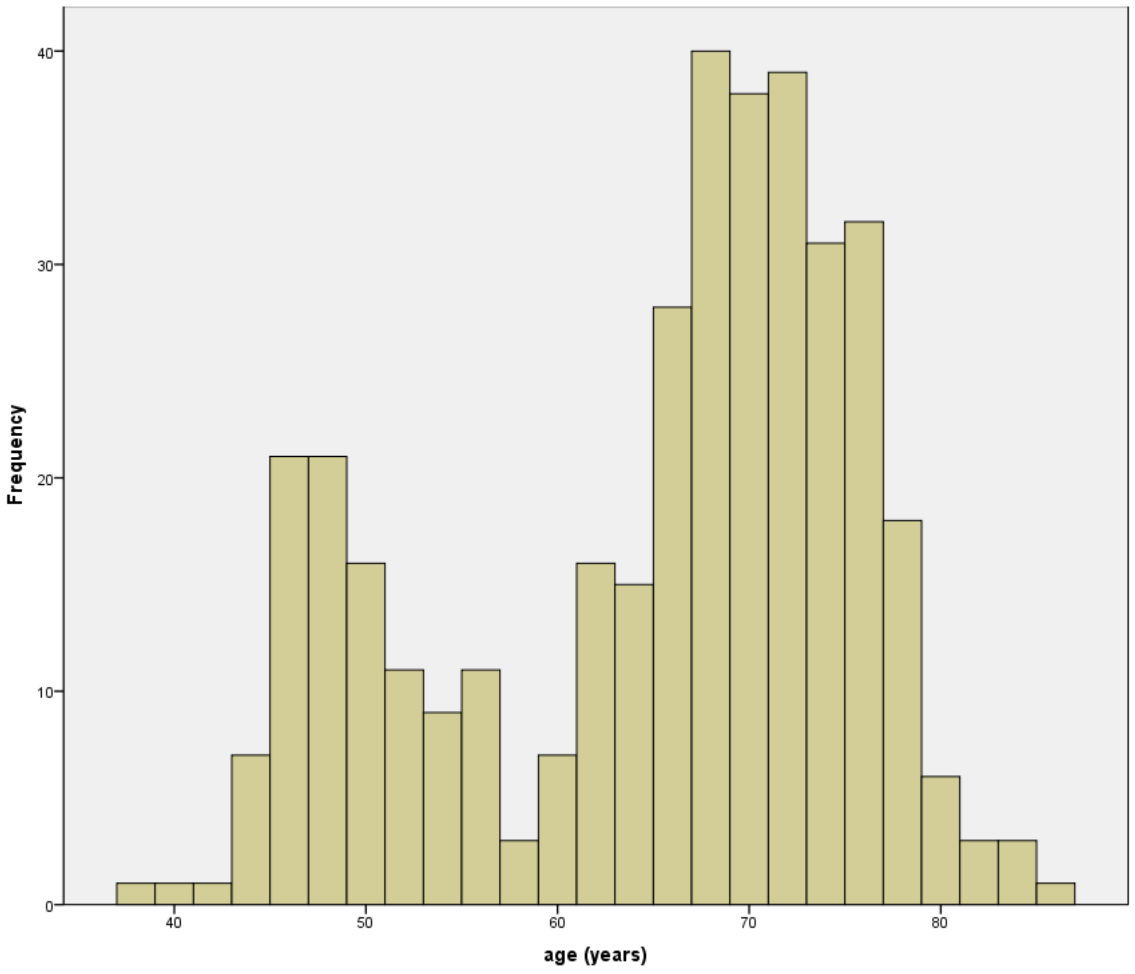

Supplementary figure 1: Distribution of age in the Study cohort

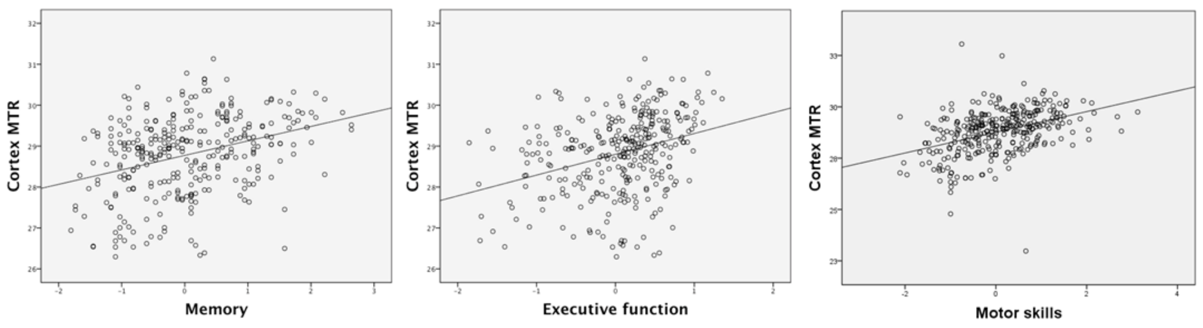

Supplementary figure 2: Scatterplots showing the correlation between cortex MTR and domain-specific neuropsychological test scores.

Abbreviations: MTR = Magnetization transfer ratio. x-axis displays z-values of neuropsychological test scores, y-axis gives MTR values of whole brain cortex.

Pearson's correlation analysis showed significant associations of cortex MTR with scores of memory ($r=0.338$, $p<0.01$), executive function ($r=0.326$, $p<0.01$) and motor skills ($r=0.472$, $p<0.01$).
