# Supplementary material for: Magnetization Transfer Ratio Relates to Cognitive Impairment in Normal Elderly
Source: Front Aging Neurosci. 2014 Sep 25;6:263. doi: 10.3389/fnagi.2014.00263 (PMC4174770; doi:10.3389/fnagi.2014.00263)
Supplement: Supplementary file 2 [file Data_Sheet1.DOCX]

|  | Males | | | | | | Females | | | | | |
| --- | --- | --- | --- | --- | --- | --- | --- | --- | --- | --- | --- | --- |
| MTR | Memory | | Executive function | | Motor skills | | Memory | | Executive function | | Motor skills | |
|  | β | p | β | p | β | p | β | p | β | p | β | p |
| **Cortex** |  |  |  |  |  |  |  |  |  |  |  |  |
| Whole brain | 0.265 | 0.007 | 0.193 | 0.002 | 0.133 | 0.154 | 0.048 | 0.660 | 0.077 | 0.203 | 0.092 | 0.314 |
| Frontal lobe | 0.253 | 0.007 | 0.180 | 0.003 | 0.107 | 0.254 | 0.028 | 0.809 | 0.077 | 0.203 | 0.090 | 0.314 |
| Parietal lobe | 0.257 | 0.007 | 0.209 | 0.000 | 0.133 | 0.154 | 0.051 | 0.623 | 0.072 | 0.212 | 0.066 | 0.462 |
| Occipital lobe | 0.250 | 0.007 | 0.216 | 0.000 | 0.133 | 0.154 | 0.063 | 0.480 | 0.075 | 0.203 | 0.084 | 0.314 |
| Temporal lobe | 0.225 | 0.013 | 0.145 | 0.024 | 0.131 | 0.154 | 0.040 | 0.707 | 0.060 | 0.284 | 0.092 | 0.314 |
| **Deep gray matter** |  |  |  |  |  |  |  |  |  |  |  |  |
| Thalamus | 0.063 | 0.416 | 0.071 | 0.061 | 0.045 | 0.440 | -0.013 | 0.809 | 0.031 | 0.316 | 0.028 | 0.566 |
| Putamen | 0.093 | 0.176 | 0.047 | 0.203 | 0.046 | 0.440 | -0.001 | 0.980 | 0.060 | 0.097 | 0.027 | 0.566 |
| Pallidum | 0.089 | 0.176 | 0.037 | 0.279 | 0.033 | 0.566 | -0.005 | 0.901 | 0.034 | 0.203 | 0.049 | 0.254 |
| Caudate nucleus | 0.044 | 0.623 | 0.048 | 0.203 | 0.010 | 0.880 | 0.024 | 0.707 | 0.076 | 0.024 | 0.039 | 0.440 |
| Amygdala | 0.072 | 0.228 | 0.050 | 0.173 | 0.038 | 0.440 | -0.010 | 0.809 | 0.011 | 0.633 | -0.002 | 0.960 |
| Accumbens nucleus | -0.020 | 0.763 | -0.006 | 0.760 | 0.010 | 0.854 | 0.005 | 0.901 | 0.043 | 0.097 | 0.016 | 0.645 |
| **NAWM** |  |  |  |  |  |  |  |  |  |  |  |  |
| Whole brain | 0.263 | 0.007 | 0.089 | 0.203 | 0.067 | 0.503 | 0.005 | 0.952 | 0.071 | 0.220 | -0.002 | 0.960 |
| Frontal lobe | 0.225 | 0.011 | 0.087 | 0.203 | 0.043 | 0.622 | -0.011 | 0.901 | 0.051 | 0.360 | 0.002 | 0.960 |
| Parietal lobe | 0.248 | 0.008 | 0.112 | 0.108 | 0.081 | 0.440 | 0.025 | 0.809 | 0.067 | 0.243 | -0.009 | 0.960 |
| Occipital lobe | 0.276 | 0.007 | 0.118 | 0.097 | 0.096 | 0.314 | 0.026 | 0.809 | 0.074 | 0.203 | 0.012 | 0.949 |
| Temporal lobe | 0.204 | 0.020 | 0.029 | 0.616 | 0.097 | 0.254 | -0.020 | 0.809 | 0.040 | 0.414 | 0.005 | 0.960 |

**Supplementary table 1.** Multivariate linear regression analysis stratified for sex*:

*Adjusted for age, years of education, vascular risk factors, cortex volume, thromboembolic infarcts, lacunes, and WMH volume.

MTR=Magnetization Transfer Ratio; NAWM=normal appearing white matter; WMH=white matter hyperintensities; p values

adjusted for multiple testing
